# Hemolysis detection for ethanol measurement in whole blood samples before centrifugation: HemCheck device evaluation

**DOI:** 10.5937/jomb0-41574

**Published:** 2023-10-27

**Authors:** Oguzhan Zengi, Meltem Boz, Beyazıt Semih Yesil, Alper Gumus

**Affiliations:** 1 Health Science University, Istanbul Basaksehir Cam and Sakura City Hospital, Department of Medical Biochemistry, Turkey

**Keywords:** hemolysis, pre-analytical phase, ethanol, point-of-care testing, quality improvement, hemoliza, preanalitička faza, etanol, testiranje na licu mesta, poboljšanje kvaliteta

## Abstract

**Background:**

As previously reported, the measurement of ethanol can also be affected by interference from hemolysis. This is a matter of concern since ethanol is widely regarded as the most commonly abused substance globally. When sample re-collection is ordered to eliminate hemolysis effects for ethanol testing, this can have unfavourable consequences for these patients. Rapid detection of hemolysed specimens would alleviate some issues associated with forensic samples. This study aimed to assess the qualitative analytical performance of a novel point-of-care testing device per the guidelines specified in CLSI-EP-12A document. HemCheck™ is a novel POCT device that qualitatively detects free-hemoglobin levels on the specimen shortly after drawing the sample.

**Methods:**

The system consists of two components. One is a cartridge with a needle that is used to transfer a small volume of whole blood from a vacuum tube to vertical and lateral flow filtration. The second component is the reader. The consumable cartridges are designed to be inserted into the reader without requiring the syringe or blood collection tube removal. A red indicator led illuminates, indicating that the sample has been hemolysed. To assess the imprecision of the method, we determined the C5-C95 interval and C50, using the Roche Cobas clinical chemistry analyser as the comparator. For this study, we utilised residual samples.

## Abstract (sr)

**Uvod:**

Kao {to je ranije objavljeno, na merenje etanola može uticati i interferencija hemolize. Ovo je zabrinjavaju}e jer se etanol smatra supstancom koja se najčešće zloupotrebljava u svetu. Kada se naloži ponovno uzimanje uzorka da bi se eliminisali efekti hemolize na testiranje etanola, to može imati nepovoljne posledice za pacijente. Brzo otkrivanje hemolizovanih uzoraka bi dalo odgovore na neke probleme povezane sa forenzičkim uzorcima. Ova studija je imala za cilj da proceni kvalitativne analitičke performanse novog ure|aja za testiranje na licu mesta, u skladu sa smernicama navedenim u dokumentu CLSI -EP12A. HemCheck™ je novi POCT uređaj koji kvalitativno detektuje nivoe slobodnog hemoglobina na uzorku ubrzo nakon uzorkovanja.

**Metode:**

Sistem se sastoji od dve komponente. Jedan je kertridž sa iglom koji se koristi za prenošenje male zapremine pune krvi iz vakuumske cevi do filtracije vertikalnog i bočnog protoka. Druga komponenta je čitač. Potrošni kertridži su dizajnirani da se umetnu u čitač bez potrebe za uklanjanjem šprica ili epruvete za prikupljanje krvi. Crvena indikatorska lampica svetli, što ukazuje da je uzorak hemolizovan. Da bismo procenili nepreciznost metode, odredili smo interval C5-C95 i C50, koristeći klinički Roche Cobas analizator kao komparator. U ovoj studiji smo koristili rezidualne uzorke.

**Rezultati:**

Naše koncentracije C50 su bile 45 mg/dL, C5-C95 30 mg/dL i 60 mg/dL, respektivno. Dve metode otkrivaju ukupno slaganje od 89,2%. Dijagnostička osetljivost i specifičnosti studije pokazala je pozitivno slaganje od 95,7% i negativno slaganje od 80,0%. Izračunata Koenova Kapa vrednost je bila 77,3%.

**Zaključak:**

Prema trenutnoj literaturi, pristrasnost nivoa etanola se može primetiti čak i sa malim koncentracijama slobodnog hemoglobina. Kada su u pitanju forenzički uzorci, ova pristrasnost je bila ključna. Na{a studija je potvrdila da HemCheck uređaj ima prihvatljive analitičke performanse, kao što je navedeno u dokumentu CLSI-EP-12A. Iako su predloženi mogući korisni uticaji inovativnog POCT uređaja za detekciju hemolize na preanalitičku fazu, mi ih nismo proučavali u ovoj studiji.

## Introduction

Hemolysis is the most frequent source of preanalytical error in clinical laboratories, accounting for nearly 60% of rejected samples [1]. Over time, the frequency of hemolysed specimens has decreased significantly due to using evacuated tube systems rather than syringes. On the other hand, hemolysed specimens still pose a significant preanalytical challenge [2]. The most common cause of hemolysed specimens is incorrect blood collection procedures [3]. Interference from hemolysis appears to affect most clinical chemistry tests [4]. As previously reported, hemolysis interference also affects ethanol measurements [5]. Ethanol is the most commonly abused substance worldwide. Ethanol test analysis is requested from medical biochemistry laboratories for medical reasons such as intoxication and forensic requirements [6]. Blood samples are valuable in both cases, and sample rejection can hinder treatment and impede forensic investigations. Ethanol measurements are frequently the subject of forensic investigations. When sample re-collection is ordered to eliminate hemolysis effects for ethanol testing, this can have unfavourable consequences for these patients [7]. Since ethanol testing frequently investigates criminal cases and traffic accidents, it requires more stringent sample quality evaluations. For ethanol analysis, chromatography is the gold standard [8]. However, since it is expensive, ethanol measurement is mainly carried out by automated enzymatic methods in routine practice. Spectrophotometric measurement of ethanol makes it more susceptible to the undesirable effects of hemolysis.

Clinical laboratory practices today are geared toward resolving hemolysed specimen challenges. For instance, rapid detection of hemolysed specimens would alleviate some issues associated with forensic samples, particularly those obtained in emergency rooms. HemCheck™ (Karlstad, Sweden AB) is a novel POCT device that qualitatively detects free-hemoglobin levels on the specimen shortly after drawing the sample [9]. The device is designed to detect hemolysis in specimens and to expedite the turnaround time for ethanol testing samples. Additionally, by utilising on-site, the cost of blood tubes and the workload of phlebotomists can be reduced.

This study aimed to assess the analytical performance of the HemCheck device and establish a user protocol to evaluate the qualitative test performance for the detection of hemolysis before centrifugation during ethanol testing.

## Material and methods

### HemCheck point of care hemolysis test device

**Figure 1:**
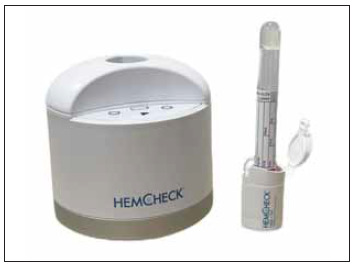
Components of the HEMCHECK measurement system

**Figure 2:**
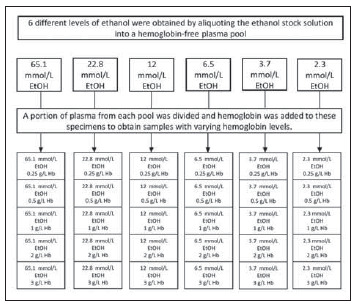
Concentration of analyte near the cut-off. The percentage of positive and negative test results from a large number of tests should change as the analyte concentration nears C50

The system consists of two components. One is a cartridge with a needle that transfers 100 μL (70–150 μL) of whole blood from a vacuum tube to vertical and lateral flow filtration. The second component is a reader, which is called H10. Consumable cartridges are inserted into the H10 reader without removing the syringe or blood collection tube. Using the detection window on the consumable cartridge, the H10 reader's CCD (charged coupled device) sensor photometrically estimates the proportion of plasma-free hemoglobin. The concentration ranges from 0 to 10 g/L free hemoglobin. The estimated plasma-free hemoglobin level is compared to a manufacturer-determined cut-off value adjusted to reflect local laboratory standards. If the result is below the defined cut-off, the user is informed via a green indicator LED signal that the sample was not hemolysed. If the result exceeds the cut-off, a red indicator led illuminates, indicating that the sample has been hemolysed (Figure 1). The local Cam and Sakura Clinical Research Ethics Committee gave the study their approval No. 330/2022. Figure 2

### Analytical performance studies

According to the CLSI-EP12-A2 document, bias and imprecision should be evaluated in qualitative tests. Imprecision studies for qualitative tests should be conducted at near cut-off concentration. Imprecision estimation would be far from the clinical decision point if these studies were conducted with low negative and high positive samples [10]. The manufacturer of HemCheck has stated that the instrument's specific cut-off value for hemoglobin concentration is >5 g/L. Ideal circumstances cannot always be obtained in an on-site area; thus, instruments should be tested for near-cut-off concentration before using qualitative testing. The current study calculated the near-cut-off value under the CLSI standard. 50% of test results would be positive, and 50% of test results would be negative at the exact cut-off concentration point. In the CLSI-EP12-A2 document, this point is designated as C_50_. An imprecision curve was plotted to present a graphical description of near-cut-off and claimed cut-off concentrations. Two sample pools were prepared with concentrations 20% above and 20% below the cut-off to verify the cut-off. Each pool is then analysed in 40 replicates to determine the per cent positive and negative results for each sample. At least 95% of measurements in the -20% and +20% pools could not be classified as positive or negative, respectively. The C5–C95 interval was then extended until 95 per cent positive and negative results were obtained.

### HemCheck vs Roche Cobas 8000

Due to the absence of universally accepted reference methods in our laboratory, the Roche Cobas-8000 hemolysis index measuring method was used as a comparator. The Roche Cobas Serum Index Gen.2 reagent (REF No: 04489365 190) was used to determine the plasma-free hemoglobin concentration. After diluting the plasma sample with saline (0.9% NaCl), absorbance measurements are taken at two different wavelengths. According to CLSI, the candidate method must be evaluated against diagnostic accuracy criteria for the highest-level comparison. Our comparative method is not used as a diagnostic accuracy criterion. Therefore, to estimate the specifications of positive and negative agreement and the kappa value, random 120 residual samples from the emergency department were used. This sample set included a broad range of results, from negatives to high positives. A whole blood sample was frozen and thawed to create the stock hemolysate. We created a range of plasma pools using the hemolysate with varying percentages of free hemoglobin. These aliquots were used in the comparison study, which included both the Roche Hemolysis index reagent and qualitative HemCheck analysis.

### Statistical analyses

EP Evaluator (Data Innovations v.12.3) and Microsoft 365 Excel were used to examine the data. To analyse the agreement and calculate the kappa value, a 2x2 consistency table was used. The bubble chart is used to compare the two methods. For non-quantitative data, it is equivalent to a scatter plot. The circle's diameter (area) is proportional to the number of specimens. The ideal chart is entirely composed of circles of a brighter colour. On the central diagonal, brightly coloured circles represent agreement. The darker-coloured circles represent points of disagreement. For qualitative comparison, darker-coloured circles indicate false positives. Cohen's Kappa: Similar to the agreement, but with the probability of the two methods agreeing by chance adjusted. Kappa values range from -100 to 100 per cent. A value of 0 indicates that there is no agreement. A value of 100% indicates complete agreement. Kappa should be well above 75%. McNemar Test for Symmetry: A test for bias – whether one method consistently produces larger values than the other. If the number of cases where X>Y is equal (within random error) to the number of cases X<Y, the method is unbiased, and the symmetry test passes. Suppose most of the differencesbetween X and Y occur when X>Y (or X<Y), the symmetry test fails.

The agreement is the proportion of all cases in which the two methods produce identical results. Related statistics for qualitative tests include the following: Positive agreement – the percentage of cases that match when the comparative method is positive is expressed as TP/(TP+FN). Negative agreement – when the comparative method is negative, the negative agreement refers to the percentage of cases that match TN/(TN+FP).

## Results

### Near cut-off (Verification of the cut-off)

Quantitative tests have manufacturer-defined cut-off values. It is a threshold value above which the result is positive and negative below which is negative.

**Figure 3:**
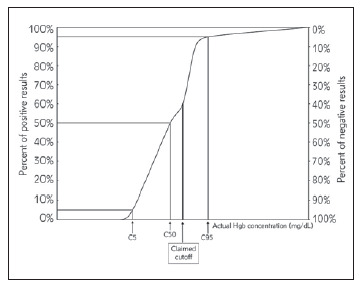
The bubble chart of the comparison of two methods. The reference or comparable method is shown on the x-axis. On the y-axis, the candidate method is displayed

Variables such as lot-to-lot variation, storage conditions, different operators, and ambient temperatures may all affect the candidate method. Because the variability exhibits distinct characteristics near the cut-off value, we should calculate the near-cut-off value for the candidate method. The manufacturer determines the cut-off concentration; users cannot change it. The manufacturer determines the cut-off value based on the test's intended use and the desired clinical specificity and sensitivity. The results would be positive 50% and negative 50% at the exact cut-off concentration, which is why this concentration point is referred to as C50 in the CLSI document. C50 concentrations may vary between laboratories. Additionally, the C50 concentration would differ according to the manufacturer-defined cut-off value. The HemCheck system has a cut-off value of >50 mg/dL for hemoglobin measurement. To verify this value, we calculated the C5-C95 interval and C50 to determine imprecision. Our C_50_ concentrations were 45 mg/dL, the C_5_–C_95_ 30 mg/dL and 60 mg/dL, respectively (Figure 3).

### Sensitivity and specificity estimation

Diagnostic sensitivity and specificity cannot be readily estimated in the common situation where the comparative method is not the criterion for diagnostic accuracy. While the same calculations are used to estimate sensitivity and specificity, the terms used are different. Rather than sensitivity and specificity, the estimates are referred to as PPA (positive per cent agreement) and NPA (negative per cent agreement) [10]. As a result, we emphasise that the estimates directly relate to candidate test agreement with the comparative method, not diagnostic accuracy. The overall agreement percentage is calculated as follows: (a: Number of results positive with both methods, b: number of results negative with both methods, c: number of results positive with the comparative method but negative with the candidate method)

The statistical analysis of the two methods reveals an overall agreement of 89.2% (CI: 82.3–93.6), 95% confidence interval was calculated using the »score« method. The positive agreement was 95.7%, while the negative agreement was 80.0%. McNemar Test for Symmetry: HemCheck < Cobas8000-H index 3 (2.5%), HemCheck > Cobas8000-H index 10 (8.3%). The symmetry test passes p=0.052 (ChiSq=3.769, 1 df). The value of p<0.05 suggests that one method is consistently »larger«. Cohen’s Kappa 77.3% (95% CI: 65.6 to 88.9%), Kappa is the portion of agreement above what is expected by chance. Kappa >75% is considered a »high« agreement.

## Discussion

In modern laboratory practice, in-vitro hemolysis is a significant source of preanalytical error, jeopardising the reliability of test results. Hemolysis has been shown to have detrimental effects on patient care, including increased turnaround times and decreased cost-effectiveness [11]. Hemolysis also has the potential to cause significant judicial and administrative complications when ethanol measurements are conducted for forensic purposes. For instance, the Turkish road traffic regulation requires that public transportation services operate with a blood alcohol concentration of zero [12]. Çat et al. [5] reported that after 1 g/L plasma Hb, the enzymatic method had a significant bias for measuring plasma ethanol; additionally, this observation contradicted manufacturer statements. According to Ustundag et al. [12], the use of the CLIA TEa limit (±25%) for blood alcohol testing is debatable for judicial decision-making. The Working Group for Forensic Toxicology recommends a bias of ±10% or less [12] [13] for ethanol analysis. The tighter tolerances necessitate stringent interference testing for blood ethanol analyses. Due to their cost effectiveness and compatibility with commonly used chemistry analysers, clinical laboratories primarily use enzymatic assays to determine blood alcohol levels. Methods not affected by hemolysis, such as headspace gas chromatography, are not frequently used in medical laboratories to determine ethanol concentration [7]. The present literature suggests that the enzymatic assays utilised to quantify blood ethanol levels are subject to interference from hemolysis. The enzyme catalase, which is found in relatively high concentrations of erythrocyte content, has been reported to interact with ADH and cause interference in ethanol measurement [14].

The manufacturer of our ethanol reagent claims that free hemoglobin levels do not impact their methodology up to 2 g/L Hb. However, this assertion contradicts the findings of Duhalde et al. [9], who reported that a free Hgb concentration of 1 g/L had a negative effect on ethanol measurement. For this reason, ensuring the preanalytical quality of ethanol blood testing specimens is critical.

HemCheck is a novel POCT device that detects hemolysed samples during the blood drawing phase. It has been known that hemolysed blood gas samples cannot be visually identified or quantified using index measurements [15]. HemCheck, according to Duhalde et al. [9], can detect hemolysis directly in blood gas samples taken at the point of care. As we mentioned above, ethanol measurements have some significant considerations in reporting issues. Rapid detection of hemolysis via POCT can also help improve the preanalytical quality of ethanol measurements. We assessed HemCheck's qualitative detection of hemolyses as well as its analytical performance when ethanol sample tubes were used. Duhalde et al. [9] found that the POC method identified hemolytic samples had a sensitivity of 80%, a specificity of 99%, and positive and negative predictive values of 89 and 98%, respectively. Our study determined that the method had a 95.7% PPA, an 80.0% NPA, and a Cohen's Kappa of 77.3%. These findings were consistent with those of Duhalde et al. [9].

**Figure 4:**
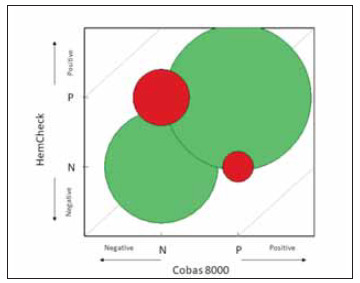
The bubble chart of the comparison of two methods. The reference or comparable method is shown on the x axis. On the y axis, the candidate method is displayed

**Figure 5:**
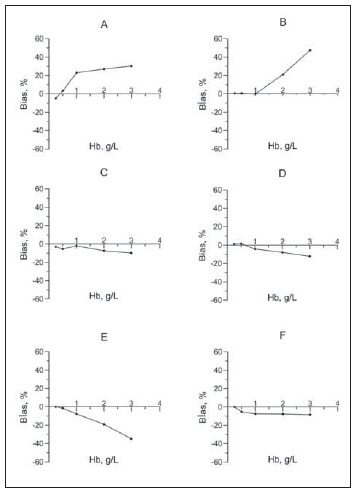
Hemolysis interferograms at various ethanol concentrations. The interferograms show how the groups’ biases are represented. (Ethanol concentrations, A: 65.1 mmol/L, B: 22.8 mmol/L, C: 12 mmol/L, D: 6.5 mmol/L, E: 3.7 mmol/L, F: 2.3 mmol/L)

**Figure 6:**
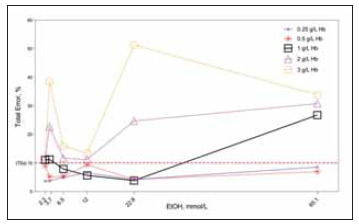
The calculated total errors of the groups are plotted. The x-axis shows different ethanol concentrations, while the y-axis shows the percentage of total error

According to our evaluation, HemCheck's analytical performance specifications were acceptable compared to a comparator method, the Roche Cobas HIL Index. When dealing with non-quantitative data like the HemCheck point of care device, the Bubble Chart is the equivalent of a scatter plot. Green circles on the central diagonal indicate agreement. The circle's area grows with the number of specimens. The ideal chart is all green. Red circles denote points of disagreement (see Figure 4) [16]. Additionally, we used Cohen's kappa to evaluate the agreement between HemCheck and the HIL index method (comparator). A Cohen's of 60%–80% indicates significant to a near-perfect agreement. We found a k value of 77.3% (95% CI: 65.6 to 88.9%), which is a remarkable performance. If the lower limit of the CI is greater than 60%, the kappa value is in excess of 60% with 95% certainty [10]. Our lower limit of the CI was 65.6%, indicating a high degree of confidence in the agreement between the two methods. Table 1 Figure 5 Figure 6

**Table 1:** When Using a Comparative Method, a 2x2 Contingency Table (the true diagnosis is unknown)

HemCheck<br>(Candidate<br>Method) N/P	COBAS-8000 HIL Index<br>(Comparative Method)<br>Cutoff: 50 mg/dL		
	Negative	Positive	Total
Negative	40	3	43
Positive	10	67	77
**Total**	**50**	**70**	**120**

In our emergency laboratory setting, we conducted an analysis of orders for blood ethanol dating back to 2021. Our analysis using H indices revealed that the percentage of hemolysed ethanol samples was 10%, which surpassed the threshold for significant bias. The majority of our hospital's ethanol requests come from traffic accidents and criminal cases; thus, our ratio of hemolysed samples may be considered high. Directly detecting hemolysed samples on the site area by phlebotomists can contribute significantly to the preanalytical phase quality. Earlier studies concur that the timely identification of hemolysed samples through the use of a POCT device is beneficial and should be an essential component of the diagnostic process to prevent potential misdiagnosis [9] [17].

This study has a limitation in that we did not investigate the regular use of HemCheck in the onsite area, which prevented us from obtaining results on the impact of HemCheck usage during the preanalytical phase. Our study was subject to an additional limitation, as we used Roche HIL index reagent to measure hemoglobin levels. Nonetheless, our results are consistent with existing literature, indicating that this method yields satisfactory outcomes compared to measurements obtained through hematology analysers.

## Conclusion

In conclusion, our study demonstrated that a novel POCT hemolysis detection device could significantly improve the quality of our preanalytical specimens, particularly forensic specimens. The device will not be cost-effective if it is used for all samples (ED, outpatient, and inpatient clinics). However, cost-effectiveness can be overlooked in the case of forensic samples due to the device's superior analytical performance.

## Dodatak

### Acknowledgement

The authors declare that they have no conflicts of interest. The article’s writing and content are solely the writers’ responsibility.

### Funding

This study was not funded by any corporation.

### Conflict of interest statement

All the authors declare that they have no conflict of interest in this work.
